# Preference of Ligneous Forages by Sheep in South-East Mali

**DOI:** 10.3390/ani15081102

**Published:** 2025-04-10

**Authors:** Mamadou Coulibaly, Drissa Coulibaly, Regina Roessler, Hawa Coulibaly, Baba Cissé, Eva Schlecht

**Affiliations:** 1 Département Sciences Animales, Institut Polytechnique Rural-Institut de Formation et de Recherche Appliquée (IPR-IFRA), Koulikoro BP 06, Mali; coulibalym017@gmail.com (M.C.); drissaycoulibaly@yahoo.fr (D.C.); coulibhawa@yahoo.fr (H.C.); babacisse260@gmail.com (B.C.); 2 Programme Bovin, Institut d’Economie Rurale (IER), Rue Mohamed V, Bamako BP 258, Mali; 3 Animal Husbandry in the Tropics and Subtropics, University of Kassel and Georg-August-Universität Göttingen, Steinstr. 19, 37213 Witzenhausen, Germany; regina.roessler@uni-kassel.de

**Keywords:** feed preference, ingestion, ligneous forage, sheep

## Abstract

In many tropical countries, woody forage plays an important role in ruminant nutrition. Five woody species commonly used in south-eastern Mali were subjected to a preference test, in order to examine their potential of being included in the rations of stall-fed sheep. A complete randomised block (Latin square) experiment with four rams was carried out during two test periods of five days each to test the appreciation of fresh and dried leaves, respectively, of the five species. On each test day, the five species were offered concomitantly to the rams for 30 min in the morning, and the parameters of their consumption along with the nutritional value were determined. The average consumption of fresh leaves was 160.9 g, 115.8 g, 66.2 g, 11.6 g, and 5.7 g per kilogram of live weight, respectively, for *Pterocarpus lucens*, *Entada africana*, *Ficus sycomorus*, *Pterocarpus erinaceus*, and *Khaya senegalensis*. For dry leaves, the consumption per kilogram of live weight averaged 69.3 g, 39.3 g, 25.0 g, 8.4 g, and 3.7 g for *P. lucens*, *F. sycomorus*, *P. erinaceus*, *K. senegalensis*, and *E. africana*, respectively. These results indicate that *P. lucens*, followed by *E. africana* when fresh, and by *F. sycomorus* when dry were the most liked species and therefore show potential to be used as a feed supplement in the rations of stall-fed sheep.

## Simple Summary

We tested the preference of the leaves of five woody fodder plants (*Entada africana*, *Ficus sycomorus*, *Khaya senegalensis*, *Pterocarpus erinaceus*, and *Pterocarpus lucens*) frequently used to feed sheep in south-eastern Mali. The results indicate that the fresh leaves of *Pterocarpus lucens*, followed by those of *Entada africana*, and the dry leaves of *Ficus sycomorus* were the most appreciated and can therefore be used as a feed supplement for stall-fed sheep.

## 1. Introduction

In sub-Saharan Africa, livestock farming contributes about 50–80% of the agricultural Gross Domestic Product (GDP). It provides food security for families and plays an important role in poverty reduction [1]. In West Africa, Mali is among the countries where livestock farming plays a major socio-economic role. However, the country’s livestock sector faces several constraints, including the scarcity and degradation of natural resources and a reduction in grazing land [2]. Currently, the diversification of the forage base for farm animals is actively discussed. On the one hand, there is a global depletion and reduction in pastures, sometimes pushing herders to utilise protected areas [3]; on the other hand, forest reclamation activities and planting valuable fodder tree species may enhance forage availability and diversity.

In Mali, the main source of ruminant feed is natural pasture vegetation, but green herbaceous fodder is only available for a short period of the year [4]. During the cool dry season (November to February), pasture vegetation remains available in sufficient quantity, but its nutritional value decreases due to the lignification and maturation of the herbaceous plants, which are transformed into “bush hay”. The quantity and quality of pasture vegetation is very low during the hot dry season (March to May) [4]; additionally, most dry pasture grasses and some cereal residues are also low in protein throughout the dry season; in this situation, woody forages can play an important role in ruminant diets [5]. However, while goats spend about 30% of their grazing time on woody species when available, sheep and cattle predominantly graze on grasses [6]. As it becomes more common to house sheep in sheds and fatten them for sale or for festivals [4], the availability of high-quality feed is critical for sheep farmers [7]. The leaves and pods of certain woody species provide quality feed for ruminants, and their use is particularly high during the dry season, when the availability and quality of other fodder resources are reduced [8,9]. Most woody forage species retain green leaves for most or all of the dry season [10]; these green leaves, along with other organs such as young twigs, inflorescences, and fruits, are rich in nutrients, especially protein, which remains high during the dry season [11]. On the one hand, the inclusion of woody feed components in the diets of goats [12] and sheep [13,14] is highly relevant but requires a sustainable use of naturally occurring woody species in accordance with the cutting techniques prescribed by the International Union for Conservation of Nature (IUCN). On the other hand, several studies have shown that woody forages contain anti-nutritional substances such as tannins and saponins, which affect their palatability and intake, as well as the performance of animals that consume them in large quantities [4,15,16,17,18].

Palatability or preference is a complex phenomenon based on specific factors. Cissé [10] defines preference as the characteristics of the plant, the animal, and the environment that stimulate selective intake by the animal in the presence of two or more forages. According to [19], palatability is related to factors inherent in the plant species that provoke a selective response from the animal, whereas preference involves the proportional choice of one plant species among two or more and is essentially behavioural. For the satisfactory performance of (stall-fed) animals, the palatability and preference of all diet components is paramount. Taking into account animal preferences should also help to optimise voluntary feed intake [20]. Methods based on the direct observation and measurement of feed intake on pasture or at the trough appear to be the most appropriate for palatability studies [21,22]. In Nigeria, [23] stated that the degree of palatability of a plant species can be determined using indices such as the ratio of biomass offered to that consumed or simply by reference to the time spent by the animal eating a particular (category of) plant species. This study aimed to identify locally used woody forage species (*Entada africana*, *Ficus sycomorus*, *Khaya senegalensis*, *Pterocarpus erinaceus*, and *Pterocarpus lucens*) that can enrich the nutritional quality of dry season rations for stall-fed sheep by evaluating their chemical composition together with animal preference, eating behaviour, and dry matter (DM) intake.

## 2. Materials and Methods

### 2.1. Study Site

This study was carried out in the livestock unit of the “Institut Polytechnique Rural de Formation et de Recherche Appliquée” (IPR-IFRA) of Katibougou, located about 3 km south of the town of Koulikoro in Mali, with the geographical coordinates 12°45′24″–12°77″05′ North, 7°33″10′–7°65′8′ West, and an altitude of 326 m above sea level. The region is situated in the Sudano-Sahelian zone, with an average annual rainfall of 900 mm (June to October) and an average annual temperature of 31 °C.

### 2.2. Experimental Animals and Woody Forages

The trial involved four intact male *Maure* breed sheep, aged between 15 and 24 months, chosen in analogy to previous preference tests with sheep [5,22] and with an average live weight of 25 ± 3.1 kg. Before the start of the experiment, the animals were treated with Ivomec^®^ against internal and external parasites. This study was ethically approved by the management of IPR-IFRA (24 February 2022).

The plant material for the preference trial was selected on the basis of a previous participatory study on farmers’ knowledge of ruminant feed preferences [24] and consisted of the leaves of *Entada africana* Guill. & Perr., *Ficus sycomorus* L., *Khaya senegalensis* Desr., *Pterocarpus erinaceus* Poir., and *Pterocarpus lucens* Lepr. ex-Guill. & Perr.

Leaves were collected by hand from the branches of five trees per species that were approximately 10 to 18 years old and growing on natural rangelands near the institute; leaves were used in both fresh and dry states to assess preference differences. For dry leaves, the material was gathered at the start of the rainy season (June–July 2022), pre-dried in the sun for 24 h, then air-dried in the shade until the weight was constant, and loosely stored in bags until testing. Fresh leaves were collected daily from the same individual trees for each species in August 2022, marking the start of the test. Each afternoon preceding a test day, fresh leaves were gathered and stored in the shade to minimise moisture loss.

### 2.3. Experimental Design and Procedures

**Figure 1:**
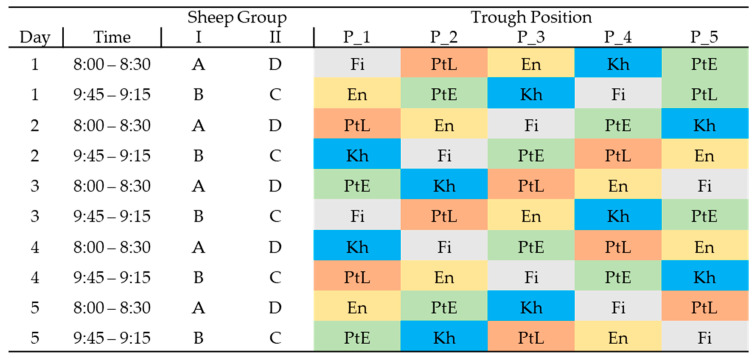
Setup of the cafeteria test: A, B, C, D are the experimental sheep. Woody forage species: *Entada africana* (En), *Ficus sycomorus* (Fi), *Khaya senegalensis* (Kh), *Pterocarpus erinaceus* (PtE), and *Pterocarpus lucens* (PtL). The same setup was used in period 1 when 650 g of fresh leaves were offered per sheep and day, and in period 2 when 200 g of dried leaves were offered per sheep and day.

The experiment followed a complete randomised block design (Latin square) with 4 sheep, numbered A to D, that were divided into two experimental groups (Figure 1). The animals were housed inside a roofed open barn, within 4 individual observation compartments, each 2.5 m long and 2.0 m wide, which allowed the visual and acoustic contact between animals. Their live weight (LW) was determined at the beginning, middle, and end of the trial after overnight fasting (about 12 h without feed) using an electronic hanging scale (Kern & Sohn GmbH, Balingen, Germany; 60 kg capacity, 0.05 kg accuracy).

The cafeteria-type preference test [22] lasted 18 days in total; these were divided into two 9-day periods, one period for fresh and one period for dry leaves. Since the experimental animals were familiar with the plant species from previous grazing on natural pastures, the pre-test phase was limited to four days per period [21]. During this phase, the sheep’s preferences for specific feed trough positions were evaluated using only the basal ration (bush hay and wheat bran; see below). During the 5 test days per period, the amount of each of the five woody species offered to each animal was weighed in the morning just before feeding. To ensure that the amount of fresh and dry leaves was approximately equal on an air-dry matter basis, 650 g of fresh leaves and 200 g of dry leaves were offered per animal and test day (electronic table scale, G&G GmbH, Kaarst, Germany; 6 kg capacity, 0.1 g accuracy). The leaves of each plant species were offered in individual feed bowls labelled with the plant species name and placed in a feed trough. The position of the leaves in each trough was changed daily to prevent the sheep from developing a preference for a particular trough position (Figure 1). Throughout the test, the animals were fed leaves before receiving the rest of their feed. For reasons of behavioural observation, sheep A and D were offered the leaves from 8:00 to 8:30 a.m., while sheep B and C received the leaves from 8:45 to 9:15 a.m. During these 30 min intervals, their feeding behaviour was observed by a person who could supervise two animals at a time, and the amount of leaves ingested per species was recorded using the method of [22] for both fresh and dry leaves. The time when the animal approached a particular trough position and the time spent feeding at that trough were exactly recorded. After 30 min, all the leaves were removed, the residues per species were weighed, and the sheep received a basal ration consisting of bush hay (ad libitum) and wheat bran (500 g animal/day, as fed), 50% of which was distributed between 9:30 and 12:00 a.m. and 50% between 1:00 and 3:00 p.m. No observations of feeding behaviour were made at this time.

After 4:15 p.m., but no later than 6 p.m., any leftovers of the basal ration were removed, and the animals were deprived of feed until the next morning, with drinking water and a mineral lick being available throughout the 24 h day.

### 2.4. Plant Sample Analysis

Samples (about 100 g) of each type of feed (fresh and dry leaves, bush hay, wheat bran) were collected daily during the two 5-day test periods, and a composite sample (100 g) was constituted for each individual feed type and test period. Samples were air-dried and ground through a 1 mm sieve prior to analysis. For each sample, all the proximate analyses were carried out in duplicate; in the Results section, however, only the values for the woody forages are displayed.

The dry matter (DM) content of the samples was determined by oven-drying at 60 °C until mass constancy was reached; a one-gram sub-sample was then dried at 105 °C for 72 h [25]. Crude ash (CA) and organic matter (OM) concentrations were determined after combustion at 550 °C overnight [25]. The nitrogen (N) concentration was determined by the Kjeldahl method [25] using a Vapodest Vap 50s apparatus (Gerhardt GmbH & Co. KG, Königswinter, Germany), and the crude protein (CP) concentration was calculated by multiplying the N concentration by a factor of 6.25. Amylase-treated neutral detergent fibre (aNDF) and acid detergent fibre (ADF), both including residual ash, as well as acid detergent lignin (ADL) were determined according to [26] using an Ankom200 semi-automatic fibre analyser (ANKOM Technology, Macedon, NY, USA). Condensed tannins (CTs) were determined by the butanol–HCl method as described by [27], and the results were expressed as leucocyanidin equivalents.

The in vitro metabolisable energy (IVME) content of the samples was estimated by measuring the gas produced during the anaerobic fermentation of the substrate according to Menke et al. [28]. The feed samples (200 mg DM) were incubated at 39 °C in triplicate on two different days in calibrated 100 mL glass syringes to which 30 mL of an incubation medium was added containing buffer solution and rumen fluid freshly collected from a rumen-fistulated cow at the University of Göttingen that was fed a standard donor animal diet. Gas production, determined by the movement of the syringe plunger, was measured over 24 h, and the correction for gas production due to the incubation medium only and the gas production of standard sample material were used to estimate the IVME as follows [29,30]:

IVME (MJ/kg DM) = 0.15 + 0.1557 × dOM + 0.0130 × CA

where GP is gas production (mL/200 mg DM), dOM is the digestibility of organic matter, and CA is the crude ash content (g/kg DM).

The dOM was calculated using the formula of Menke et al. [30]:

dOM = 15.38 + 0.8453 × GP + 0.0595 × CP + 0.0675 × CA

where GP is gas production (mL/200 mg DM), CP is the crude protein content (g/kg DM), and CA is the crude ash content (g/kg DM).

### 2.5. Data Analysis

All the data were entered into Microsoft Excel 2019 for the calculation of various variables. The amount of leaf fresh matter (FM) and dry matter (DM) ingested per unit live weight (kg LW) was calculated from the amount of leaves offered and refused during the daily 30 min periods. In addition, the cumulative time (in seconds) spent eating a particular leaf type was calculated to obtain the total consumption time. The preference coefficient for each plant species and leaf state (fresh, dry) was calculated as the ratio of the amount of leaves consumed per species to the total amount of leaves consumed across all species.

For the nutritional quality of the leaves, only descriptive statistics were calculated. The statistical analyses of the amount of leaves ingested during the 30 min observation interval, the total consumption time, and the preference coefficient as well as the figure production were performed in R version 4.1.3, using additional functions provided in the R packages version 4.1.3 *lme4* [31], *lmer* test [32], and *boxplot* [33]. For the comparison of woody forage means within leaf types, the non-parametric Kruskal–Wallis test [34] followed by the Wilcoxon post hoc test [35] were used, with a significance threshold of *p* < 0.05.

## 3. Results

### 3.1. Chemical Composition of Leaves

*E. africana* and *P. lucens* had similarly high OM concentrations (g/kg DM) of 940.7 and 940.6, respectively (Table 1), while *F. sycomorus* had the lowest value (780.9). The CP concentration (g/kg DM) was lowest for *K. senegalensis* (125.4) and highest for *E. africana* (214.6). The CT content (g/kg DM) was highly variable, being highest in *K. senegalensis* (41.0) and *P. lucens* (35.6) and lowest in *E. africana* (6.9). The cell wall components (aNDF, ADF, ADL) were high in *P. lucens* and *P. erinaceus* but low in *K. senegalensis* and *E. africana*. With 20.1 MJ/kg DM, *E. africana* had a higher IVME content than all the other species, while *K. senegalensis* had the lowest energy content (10.3 MJ/kg DM).

### 3.2. Quantitative Intake of Leaves

The results of the test for trough positions during the two 4-day adaptation periods showed no significant location preference (*p* > 0.05) among the individual sheep. Additionally, the average feeding time of the sheep at each position was similar.

The average intake of fresh leaves (g FM/kg LW) during the 30 min observation interval was higher (*p* < 0.05) for *P. lucens* (160.9) than for *E. africana* and *F. sycomorus* (Table 2), while *K. senegalensis* was the species with the lowest fresh leaf intake (5.7). The average consumption of dry leaves (g DM/kg LW) varied from 3.7 (*E. africana*) to 69.2 *(P. lucens).* Except for *F. sycomorus*, the difference in consumption between *P. lucens* and the other forage species was highly significant (*p* < 0.001). The Wilcoxon pairwise comparison showed no significant difference between *P. lucens* and *F. sycomorus* on the one hand, and between *E. africana*, *P. erinaceus*, and *K. senegalensis* on the other (Table 2).

### 3.3. Consumption Time and Preference of Woody Species

**Figure 2:**
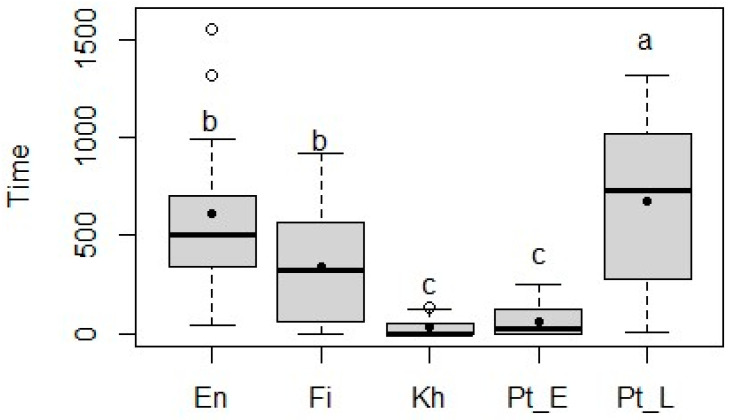
Time (seconds) taken by sheep to ingest the fresh leaves of *Entada africana* (En), *Ficus sycomorus* (Fi), *Khaya senegalensis* (Kh), *Pterocarpus erinaceus* (Pt_E), and *Pterocarpus lucens* (Pt_L) during a 30 min period. The lower and upper limits of the boxes represent the lower and upper quartiles, the horizontal line indicates the median, the black dot indicates the mean, and the lower and upper whiskers indicate the minimum and maximum. The outliers are marked by the white dots. The different superscripts (a–c) indicate a significant difference (*p* < 0.05) between means.

The mean consumption times for fresh leaves ranged from 59 ± 75.3 s for *P. erinaceus* to 680 ± 418.9 s for *P. lucens* (Figure 2). The high mean leaf consumption times during the 30 min observation interval indicated that the most preferred species were *P. lucens* and *E. africana*, while *K. senegalensis* and *P. erinaceus* had similarly low mean consumption times.

**Figure 3:**
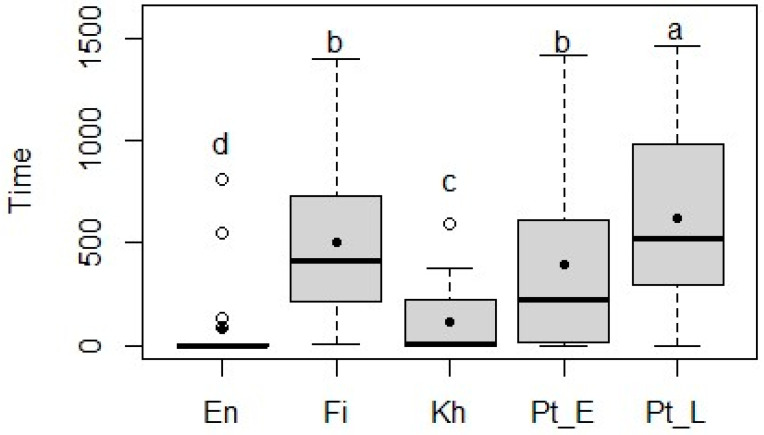
Time (seconds) taken by sheep to ingest the dry leaves of *Entada africana* (En), *Ficus sycomorus* (Fi), *Khaya senegalensis* (Kh), *Pterocarpus erinaceus* (Pt_E), and *Pterocarpus lucens* (Pt_L) during a 30 min period. The box limits represent quartiles; the horizontal line is the median, the black dot is the mean, and the whiskers show the minimum and maximum. The white dots mark the outliers. The superscripts (a–d) indicate significant differences (*p* < 0.05) between means.

For dry leaves, the mean consumption time varied from 40 ± 212.2 s for *E. africana* to 251 ± 427.6 s for *P. lucens* (Figure 3). Next to *P. lucens*, the dry leaves of *F. sycomorus* had a high total consumption time, while *E. africana* was the species with the lowest consumption time, followed by *K. senegalensis*. Statistical analysis revealed significant differences between the woody species (*p* < 0.001) for the mean consumption time of fresh and dry leaves, respectively. Additionally, the Wilcoxon pairwise comparison indicated significant differences between *P. lucens* and all the other species, in both fresh and dry states.

**Figure 4:**
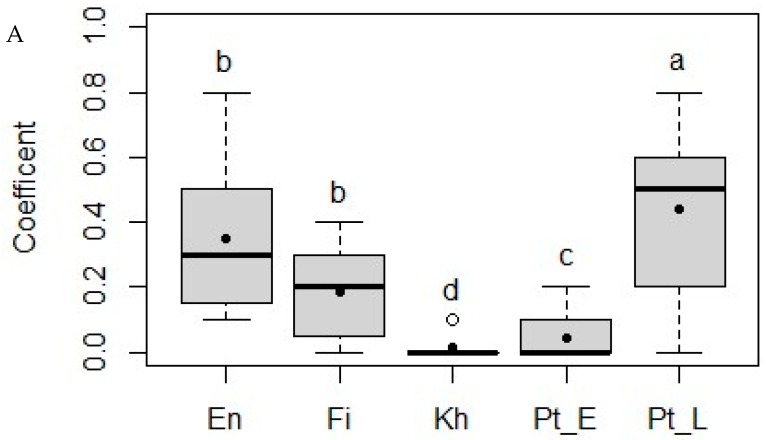

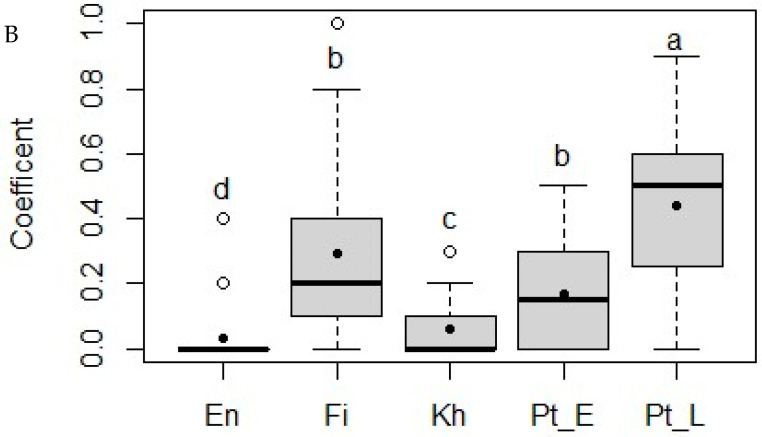
Preference coefficient for five different woody species by sheep: *Entada africana* (En), *Ficus sycomorus* (Fi), *Khaya senegalensis* (Kh), *Pterocarpus erinaceus* (Pt_E), and *Pterocarpus lucens* (Pt_L) in fresh (**A**) and dry (**B**) states. The box limits represent quartiles, the horizontal line is the median, the black dot is the mean, and whiskers show the minimum and maximum. The white dots mark the outliers. The superscripts (a–d) indicate significant differences (*p* < 0.05) between means.

To rank the woody species according to the sheep’s preference, the preference coefficient was calculated (Figure 4). The data indicated that the preference coefficients varied between the woody species in both the fresh and dry states. In both conditions, *P. lucens* was the most preferred species. However, while *K. senegalensis* was the least preferred in the fresh state, *E. africana* was the least preferred in the dry state.

## 4. Discussion

### 4.1. Nutritional Quality of Woody Forage Species

Although the leaves collected, fed, and analysed in this study were a mix collected from five different trees per species in just one season and year, they differed in their chemical composition at the species level. This has also been reported from northern Benin [36] and north-eastern Nigeria [23]. *K. senegalensis* and *P. erinaceus* showed high dry matter concentrations, while the lowest value was found in *F. sycomorus*, confirming the results of [4] in south-eastern Mali. While the leaves’ DM concentrations in our study were similar to those reported in Mali [4], they were higher than those found by [37] in the Ngouye agro-pastoral zone of Senegal. Potential factors contributing to these variations include the soil type, climatic and intra-species differences, as well as harvesting time.

Studies conducted in Burkina Faso [38] and northern Benin [36] showed that CP is an indicator of the palatability and digestibility of green fodder. Therefore, the CP content is an important factor in assessing the nutritional value of forages. In our study, the CP content of all browse species exceeded the threshold of 80 g CP/kg DM, below which appetite and feed intake are reduced [38]. The CP concentrations of the studied leaves are thus within the range required for proper rumen function and the adequate feeding of ruminants [26,39,40] and are higher than those obtained by [38] in Burkina Faso for the same woody species. The present CP content of *E. africana* is also higher than the values obtained by [41] in Tchad and [39] in the Negev desert in Israel but close to the values reported by other authors for African browse species, in particular [38,41,42] in the northern Sudanian region of Burkina Faso. The high CP contents obtained for *E. africana*, *P. erinaceus* and *P. lucens* can be explained by their ability to fix atmospheric nitrogen [43]. This favours the use of these species as protein supplements for poor-quality forages and fibrous by-products [43]. However, anti-nutritional factors such as phenolic compounds and especially tannins are likely to limit CP intake and digestibility [38].

The content of metabolisable energy is very important as it determines the intake and utilisation of a feed [44]. Since the energy value of a feed is related to its digestibility [45], the high IVME content of *E. africana* (20 MJ/kg DM) also indicates a good digestibility of its organic matter. Although *K. senegalensis* had the lowest IVME (10 MJ/kg DM), all the IVME values recorded in the present study were higher than those found in other studies from the region [36,45,46].

In addition to energy content and digestibility, the amount of feed voluntarily ingested determines its contribution to nutrition [44]. Tannins can reduce the palatability of leaves by causing an astringent sensation in the mouth of animals [47]. *K. senegalensis* leaves had a high CT content, which explains their low preference. Despite having the second highest CT content, *P. lucens* leaves had the highest preference coefficient. It is important to note that in addition to the analytically determinable CT content, their biological activity also influences their effect on animals [44]. A study conducted in Mali [4] reported that the concentration of tannins affecting palatability and digestibility in cattle and sheep was over 5%, which is higher than the CT concentrations in the studied leaves, which were all below 4.5% in DM.

Compared to *Andropogon gayanus* hay (78% NDF in DM) and bush hay (81% NDF in DM), which are the poor-quality forages [48] on which a majority of small ruminants rely as their basic feed in the dry season, the aNDF content of the leaves in our study was low, ranging from 52% to 61% in DM. The NDF and ADF contents of woody forages’ leaves are generally lower than those of herbaceous plants, even when the latter are green [43]. While the leaves of *Sterculia setigera* investigated in our study area contained 49% to 50% NDF in the dry matter [49], which is similar to the aNDF content of the browse species investigated in the present study, *Acacia nilotica* leaves were found to be less fibrous, containing only 36% NDF in the dry matter [50].

### 4.2. Preference of Woody Forages by Sheep

Understanding the feed selection behaviour of sheep when presented with several forages is crucial for determining how the animals choose and consume different plant species. Sheep exhibit clear food preferences, influenced by factors such as palatability, nutrient content, and the presence of secondary compounds like tannins, which can affect acceptability [4,22].

The amount of leaves ingested per unit of live weight was high for *P. lucens* and low for *K. senegalensis*, regardless of their state, although intake was higher for dry than for fresh leaves. The total consumption time and preference coefficients for the woody forage species also varied, and there was a relationship between the consumption time of a species, the amount ingested, and the preference coefficient: the more the sheep preferred the species, the longer it was consumed during the 30 min observation interval, and the greater the amount consumed.

The influence of the leaves’ chemical composition on feed intake was pronounced for *K. senegalensis*, which was consumed less, possibly because of its high CT content. However, *P. lucens*, which ranked second in terms of CT concentration, was the most consumed species in the present study, even in the dry state, suggesting that CTs were not the only factor influencing feed preference. In sheep, as in cattle, the voluntary daily DM intake decreased linearly with increasing NDF content [4]. This was not confirmed in our cafeteria trial, where *P. lucens* had the highest NDF concentration but was the most consumed species, accounting for 26.9% of the average daily intake of leaves during the 30 min of daily offer. According to [51], herbivores do not graze indiscriminately but have strong feed preferences. Their feed choices are primarily influenced by the nutritional value and toxin content of the plants. Several authors [52,53] have also mentioned the sorting behaviour of tropical sheep as a crucial parameter in voluntary feed intake [21,54,55].

## 5. Conclusions

The preference test proved to be an effective method of classifying the sheep’s dietary preferences with respect to the woody plants considered. The results show that *Pterocarpus lucens* is the most preferred species, both fresh and dried. When fresh, it was followed by *Entada africana* and *Ficus sycomorus*. In a dried state, *Ficus sycomorus* and *Pterocarpus erinaceus* ranked second and third. *Khaya senegalensis* was identified as the least palatable species, irrespective of its physical condition. Since the preferred species were also found to have high crude protein concentrations and energy values, farmers are advised to collect, dry, and stock the green leaves of *Pterocarpus lucens* and *Ficus sycomorus* as a dry season supplement feed for stall-fed sheep. Given that *P. erinaceus* is an endangered species listed in CITES Appendix II, farmers should be discouraged from (over)-using this tree species, despite its high value as a fodder tree based on sheep’s preferences and farmers’ perceptions.

## Figures and Tables

**Table 1:** Chemical composition of leaves of ligneous species (one composite sample of leaves of five individual trees per species, analysed in duplicate).

Plant Species	Chemical Component ^1^ (g/kg DM)							MJ/kg DM
	DM ^2^	OM	CP	CT	aNDF	ADF	ADL	IVME
*Entada africana*	457.1	940.7	214.6	6.9	516.8	392.6	185.1	20.1
*Ficus sycomorus*	428.3	780.9	129.5	11.4	537.0	378.0	134.6	11.4
*Khaya senegalensis*	579.1	910.3	125.4	41.0	516.6	375.1	146.6	10.3
*Pterocarpus erinaceus*	561.3	930.8	174.8	20.7	613.3	404.4	152.3	14.8
*Pterocarpus lucens*	522.3	940.6	152.3	35.6	614.0	455.5	247.1	11.7

^1^ DM: dry matter; OM: organic matter; CP: crude protein; CT: condensed tannins; aNDF: amylase-treated neutral detergent fibre; ADF: acid detergent fibre; ADL: acid detergent lignin; IVME: in vitro metabolisable energy, determined via triplicate incubation on 2 different days. ^2^ The average DM is the product of the air-dried matter (obtained after shade-drying to weight constancy and subsequent oven-drying at 60 °C) and residual dry matter (determined through oven-drying at 105 °C) of the composite leaf samples collected from 5 individual trees per species. DM is expressed in g/kg fresh matter.

**Table 2:** Mean voluntary intake during 30 min per day of fresh and dry leaves of ligneous species by sheep. Values are means of 4 sheep and 5 test days.

Plant Species	Fresh Leaves (g FM/kg LW)	Dry Leaves (g DM/kg LW)
*Entada africana*	115.8 ^a^	3.7 ^b^
*Ficus sycomorus*	66.2 ^a^	39.4 ^ab^
*Khaya senegalensis*	5.7 ^c^	8.3 ^b^
*Pterocarpus erinaceus*	11.6 ^b^	25.2 ^b^
*Pterocarpus lucens*	160.9 ^a^	69.2 ^a^
SEM	16.43	6.58
*p*-value	0.001	0.001

Different superscript letters after the means indicate a significant difference between woody species. DM: dry matter; FM: fresh matter; SEM: standard error of mean.

## Data Availability

For scientific purposes, the data presented in this study are available on request from the corresponding author.
